# The Evaluation of a Clinical Decision Support Tool Using Natural Language Processing to Screen Hospitalized Adults for Unhealthy Substance Use: Protocol for a Quasi-Experimental Design

**DOI:** 10.2196/42971

**Published:** 2022-12-19

**Authors:** Cara Joyce, Talar W Markossian, Jenna Nikolaides, Elisabeth Ramsey, Hale M Thompson, Juan C Rojas, Brihat Sharma, Dmitriy Dligach, Madeline K Oguss, Richard S Cooper, Majid Afshar

**Affiliations:** 1 Department of Computer Science Loyola University Chicago Chicago, IL United States; 2 Department of Public Health Sciences Loyola University Chicago Maywood, IL United States; 3 Department of Psychiatry Rush University Medical Center Chicago, IL United States; 4 Department of Medicine University of Wisconsin-Madison Madison, WI United States

**Keywords:** substance misuse, artificial intelligence, natural language processing, clinical decision support, study protocol

## Abstract

**Background:**

Automated and data-driven methods for screening using natural language processing (NLP) and machine learning may replace resource-intensive manual approaches in the usual care of patients hospitalized with conditions related to unhealthy substance use. The rigorous evaluation of tools that use artificial intelligence (AI) is necessary to demonstrate effectiveness before system-wide implementation. An NLP tool to use routinely collected data in the electronic health record was previously validated for diagnostic accuracy in a retrospective study for screening unhealthy substance use. Our next step is a noninferiority design incorporated into a research protocol for clinical implementation with prospective evaluation of clinical effectiveness in a large health system.

**Objective:**

This study aims to provide a study protocol to evaluate health outcomes and the costs and benefits of an AI-driven automated screener compared to manual human screening for unhealthy substance use.

**Methods:**

A pre-post design is proposed to evaluate 12 months of manual screening followed by 12 months of automated screening across surgical and medical wards at a single medical center. The preintervention period consists of usual care with manual screening by nurses and social workers and referrals to a multidisciplinary Substance Use Intervention Team (SUIT). Facilitated by a NLP pipeline in the postintervention period, clinical notes from the first 24 hours of hospitalization will be processed and scored by a machine learning model, and the SUIT will be similarly alerted to patients who flagged positive for substance misuse. Flowsheets within the electronic health record have been updated to capture rates of interventions for the primary outcome (brief intervention/motivational interviewing, medication-assisted treatment, naloxone dispensing, and referral to outpatient care). Effectiveness in terms of patient outcomes will be determined by noninferior rates of interventions (primary outcome), as well as rates of readmission within 6 months, average time to consult, and discharge rates against medical advice (secondary outcomes) in the postintervention period by a SUIT compared to the preintervention period. A separate analysis will be performed to assess the costs and benefits to the health system by using automated screening. Changes from the pre- to postintervention period will be assessed in covariate-adjusted generalized linear mixed-effects models.

**Results:**

The study will begin in September 2022. Monthly data monitoring and Data Safety Monitoring Board reporting are scheduled every 6 months throughout the study period. We anticipate reporting final results by June 2025.

**Conclusions:**

The use of augmented intelligence for clinical decision support is growing with an increasing number of AI tools. We provide a research protocol for prospective evaluation of an automated NLP system for screening unhealthy substance use using a noninferiority design to demonstrate comprehensive screening that may be as effective as manual screening but less costly via automated solutions.

**Trial Registration:**

ClinicalTrials.gov NCT03833804; https://clinicaltrials.gov/ct2/show/NCT03833804

**International Registered Report Identifier (IRRID):**

DERR1-10.2196/42971

## Introduction

The COVID-19 pandemic has exposed major gaps in health care delivery with limited resources and staffing. In 2020, deaths related to drug overdose reached an all-time high with a record 93,000 deaths nationwide during the pandemic year [1]. The number of substance use–related hospital visits outpaces visits for heart disease and respiratory failure [2]. Despite the recommendations from the US Preventive Services Task Force for Unhealthy Drug Use Screening [3], hospital screening rates remain low, with detection rates around 50% [4]. Manual screening efforts within busy hospital settings impose staffing requirements and administrative burdens, with the corresponding missed opportunities to prioritize care for the most vulnerable patients.

The prevalence of unhealthy substance use (nonmedical use of opioids or benzodiazepines, illicit drugs, or alcohol) in hospitalized patients is estimated to be 15% to 25%, far exceeding that of the general population, and the hospital setting is an important touchpoint for engaging patients [5,6]. Hospitals currently screening for unhealthy substance use need better approaches to identifying and treating patients, with less than a quarter of patients with a substance use disorder receiving treatment [7]. During the COVID-19 pandemic, screening efforts became even more challenging with changes in workflow and the reallocation of resources that further reduced manual screening rates [8]. Meanwhile, substance misuse ranks second among principal diagnoses for unplanned 7-day hospital readmission rates [7,9].

As of 2017, over 80% of hospitals in the United States have adopted an electronic health record (EHR) system [10]. Clinical decision support (CDS) and intelligent data-driven alerts are now part of federal incentive programs for meaningful use [11]. With access to EHR data and financial incentives to improve quality care, hospitals are increasingly well equipped to leverage computational resources to improve screening efforts via automated solutions [12,13]. The potential in digital phenotyping for substance use identification and treatment is real [14], but few pragmatic studies have been implemented to examine their effectiveness. Prior studies have demonstrated that the EHR contains information needed to identify cases of substance use [15,16]. However, leveraging data-driven methods with artificial intelligence (AI) and automating screening approaches remain in their infancy [17].

Although information about substance use is routinely recorded in providers’ intake notes in the EHR, it is neither organized nor prioritized during routine care for CDS [18]. Automated, data-driven solutions with natural language processing (NLP) can automatically extract important risk factors from clinical notes [19]. The computational methods of NLP derive semantic features from clinical notes, from which machine learning models can predict substance misuse. We previously published and made publicly available an NLP screening tool for different types of substance misuse [8]. During the validation of the algorithm, we achieved sensitivity and specificity greater than 85% using a convolutional neural network (CNN) from clinical notes with a false negative rate of less than 5% to screen for unhealthy alcohol use, unhealthy opioid use, and unhealthy nonopioid drug use (ie, cocaine and amphetamines).

In the advent of the digital era of AI in medicine, machine learning classifiers and AI-driven models are now being developed at an exponential pace. However, very few NLP systems have been translated into real-world clinical contexts with rigorous evaluation [20]. We provide a study protocol on one of the first NLP-driven solutions using our validated algorithm with the hypothesis that we can achieve a comprehensive and automated screening system that reduces workforce resources without compromising effectiveness. To test our hypothesis, we plan to examine health outcomes in a noninferiority design coupled with a cost-effectiveness analysis. More specifically, we propose a pre-post segmented regression analysis to evaluate the effectiveness of the automated screening tool in maintaining or increasing (1-tailed test) the proportion of patients who screened positive and received any of a composite group of interventions compared to usual care (eg, interviewer-administered screening).

## Methods

### Setting and Study Design

The study will be implemented at Rush University Medical Center (RUMC) across the surgical and medical hospital inpatient wards. This prospective evaluation will target all adult (18-89 years of age) hospitalizations over a 24-month period (12 months of usual care with manual screening and 12 months under the implementation of automated screening) and an additional 6-month follow-up period for secondary outcomes. We will use pre-post segmented regression analysis with noninferiority hypothesis testing to evaluate the impact of the substance misuse classifier compared to usual care. The trial is registered at ClinicalTrials.gov (NCT03833804).

### Preintervention Period: Usual Care With Manual Screening

In 2017, RUMC launched a multidisciplinary Substance Use Intervention Team (SUIT) to address the opioid epidemic through a universal screening and Screening, Brief Intervention, and Referral to Treatment (SBIRT) program in the hospital [21]. Screening, intervention flow sheets, and consult order sets were built into EHR-driven workflows for inpatient nurses and social workers. Leveraging the EHR infrastructure, the manual screening by nurses and social workers was driven by four key components: (1) a single workflow that connects the nursing and social work navigators and allows both disciplines to document screening information into a common flowsheet in the EHR; (2) a status column in the unit patient list where the social work team indicates the current stage of the intervention for each patient; (3) a consult order to addiction medicine that operates within a work queue managed by the SUIT; and (4) a flowsheet for the SUIT to document the details of the intervention. Specifically, if patients reported positive to the universal manual screening during the rooming process, an indicator in the substance use column would update to signal a social worker to conduct a full manual screening with the Alcohol Use Disorders Identification Test (AUDIT) and/or Drug Abuse Screening Tool (DAST). As part of usual care and in the preintervention period, RUMC will perform an initial 2-question universal screening for alcohol (5 or more drinks for men and 4 or more drinks for women) and drugs (any illicit drug use in the past year). Those who screen positive will receive a full screening with the 10-item AUDIT [22] or 10-item DAST [23]. Once completed, the social worker may provide a brief motivational interviewing intervention for an AUDIT score above 4 or DAST score above 1. For higher risk scores, the social worker may recommend a consult to the SUIT for addiction services. Alternatively, primary teams will be able to consult the SUIT directly, at which time, AUDITs and DASTs will be performed by the SUIT themselves. The consulting team determines with the patient whether to initiate medication and linkage to outpatient services upon discharge. If ready, patients may begin medication and, upon discharge, receive individual and group psychotherapy, case management, and continued medication treatment at an outpatient addiction medicine clinic.

### Postintervention Period: AI-Assisted Screening

We previously published a substance misuse screening tool using NLP and machine learning from the clinical notes, Substance Misuse Algorithm for Referral to Treatment using Artificial Intelligence (SMART-AI) [8]. SMART-AI was developed on hospitalized RUMC patients between October 1, 2017, and December 31, 2019, with temporal validation between January 1, 2020, and December 31, 2020. In hospitalized patients, the SMART-AI CNN used the first 24 hours of EHR notes to identify and screen for multiple types of unhealthy substance use (unhealthy alcohol use, unhealthy opioid use, and unhealthy nonopioid drug use). Temporal validation of the classifier during the COVID-19 pandemic demonstrated a mean area under the receiver operating characteristic curve of 0.97 (95% CI 0.96-0.98) and a mean area under the precision-recall curve of 0.69 (95% CI 0.64-0.74) for the different types of unhealthy substance use. The number needed to evaluate (NNE) on positive screenings to identify a true positive was 1.5 for unhealthy alcohol use, 1.3 for unhealthy opioid use, and 2.6 for unhealthy nonopioid drug use. This created 39, 26, and 16 alerts per 1000 hospitalized patients for each group, respectively. This was deemed an acceptable workload by the SUIT clinical care team. In the intervention period, the manual screening performed by nurses and social workers will be replaced with SMART-AI.

The EHR system at RUMC is provided through Epic (Epic Systems Corporation). We designed an approach to collect notes from the first 24 hours of hospitalization from Epic, which is how SMART-AI was originally developed and validated [8]. With an average time of 1.6 days from a patient’s admission to receipt of a SUIT consultation during usual care, we anticipate this is sufficient time for the automated screener to operate and clinical interventions to occur after admission notes are collected.

During the postintervention period, an alert will run every 24 hours after a nightly data extraction from the front-end EHR (Epic) into the back-end data warehouse (Clarity) at RUMC. SMART-AI will operate using the daily EHR notes collected in the data warehouse that are preprocessed through an NLP engine and fed into the SMART-AI machine learning model in a Microsoft Azure cloud computing environment. The output classifications for screen-positive cases will be published as reports routed through a secure environment for viewing. At RUMC, the patients who flagged positive for substance misuse will be reported with an encrypted email routed to the SUIT provider each morning, after the server is refreshed with the last 24 hours of data.

### NLP Pipeline

Linguistic preprocessing of the EHR to extract clinical information from unstructured text will be managed via an open-source software called the Clinical Text and Knowledge Extraction System (cTAKES; version 4.0) [24]. cTAKES processes clinical notes; identifies types of clinical named entities such as drugs, diseases/disorders, signs/symptoms, anatomical sites, and procedures; and maps them to concepts from the National Library of Medicine's Universal Medical Language System (UMLS) Metathesaurus. cTAKES is a modular pipeline that first breaks the EHR note into tokens and sentences. Next, it annotates the word tokens with parts-of-speech tags (eg, noun and adjective). Third, candidate phrases are formed and matched to a dictionary of medical concepts sourced from the UMLS. Mappings convert the raw text to standardized medical terminologies such as SNOMED CT and RxNORM, using concept codes from the UMLS called Concept Unique Identifiers (CUIs). The text spans from the EHR notes are ultimately transformed into sequences of CUIs representing UMLS-named entity mentions (diseases, symptoms, anatomy, and procedures). For instance, “heroin use” is assigned “C0600241” as its CUI and is a separate CUI from “history of heroin use,” which is “C3266350.”

The sequences of CUIs from the notes collected in the first 24 hours of hospitalization are concatenated into a single document and converted into sequences of dense vectors known as CUI embeddings, which in turn serve as the input layer to SMART-AI, a multilabel CNN. All SNOMED CT and RxNORM CUIs mapped from the notes are available to the model as 300-dimensional CUI embeddings. There is no limitation to the number of CUIs to be fed into the model, which is an advantage over pretrained language model transformers that commonly have a token limitation. SMART-AI will provide the final output classification for screen-positive cases for unhealthy alcohol, opioid, and/or nonopioid drug use. We will use a cutoff of 0.05 on the predicted probabilities for each substance use label because this provided the best test characteristics in the validation study [8]. The previously trained and validated CNN model for SMART-AI is available on Github [25], and more technical details about the model are detailed in the development and validation study [8]. No changes were made to the implementation of this protocol.

### Data Collection and Management

The clinical SUIT has previously established outcome data collection methods using the EHR [26,27]. Specific to this study, flowsheets within the EHR have been updated with hospital operations to capture additional SUIT consult parameters (Table 1). Preconsult information includes the consult modality (inpatient screen, prior patient of SUIT, ad hoc, and emergency department–initiated), the consult reason (evaluation for treatment initiation, continuity for medication-assisted treatment [MAT] maintenance, and acute care adjustment of MAT), and inpatient screen exemption reason (intoxication/overdose, withdrawal, and related physical ailment). Additionally, the post-SUIT consult disposition (complete consult, patient refusal, and incomplete consult) will be another recorded parameter within the EHR. Beginning in the preintervention period, the data will be extracted monthly from the EHR to monitor quality and completeness and will be presented biannually at scheduled Data Safety Monitoring Board (DSMB) meetings.

**Table 1:** Components of the intervention electronic health record data capture.

Component			Description
**Composite outcome**			
	Brief intervention/motivational interviewing	A social worker provides a brief intervention using motivational interviewing for alcohol or stimulant use	
	Naloxone dispensing	Prescription, free kit from clinic, or order for home kit	
	MAT^a^	Buprenorphine, methadone, or naltrexone (OUD^b^); acamprosate, gabapentin, or disulfiram (AUD^c^)	
	Addition consult	Consultation team includes specialists from emergency medicine, psychiatry, toxicology, social work, and pharmacology	
	Referral to outpatient treatment	Individual or group psychotherapy, case management, and continued MAT	
**Consult characteristics**			
	Consult modality	Inpatient screen, established patient, ad hoc, and emergency department–initiated	
	Consult reason	Evaluation for initial treatment, MAT maintenance, MAT adjustment, not substance use–related, and non-MAT disposition planning	
	Inpatient screen exemption	Intoxication/overdose, withdrawal, and related physical ailment (endocarditis, alcoholic cirrhosis, and acute psychosis)	
	SUIT^d^ provider role	Consultant, supporting other service, and curbside	
	Disposition	Incomplete, patient refusal, patient discharged, and completed consult	

^a^MAT: medication-assisted treatment.

^b^AUD: alcohol use disorder.

^c^OUD: opioid use disorder.

^d^SUIT: Substance Use Intervention Team.

### Primary and Secondary Outcomes

The primary outcome is the count of patients who had an addiction consult and received the composite intervention of any of the following: (1) naloxone dispensing (prescription, free kit from clinic, or order for home kit); (2) MAT (buprenorphine, methadone, naltrexone, acamprosate, gabapentin, or disulfiram); (3) referral to outpatient treatment; and/or (4) brief intervention/motivational interviewing for substance misuse. Each component will be indicated separately in the EHR flowsheet for the hospital encounter (Table 1).

The secondary outcome is all-cause rehospitalizations following 6 months from the index hospital encounter. Further exploratory outcomes include each component of the primary outcome analyzed separately: naloxone dispensing, MAT, referral to outpatient treatment, and brief intervention/motivational interviewing for unhealthy substance use. We will also characterize the time to consult and discharge rates against medical advice (AMA).

### Analysis Plan

Descriptive statistics will be reported for demographic and clinical variables stratified by pre- and postintervention periods. Outcomes will be compared by time period (pre- vs postintervention) using generalized linear mixed effects models (GLMMs), which will allow for appropriate modeling of the different dependent variables, including continuous, count, or categorical, and to include random effects to account for correlated data due to patients with multiple index hospitalizations. For the primary end point, we will first plot the proportion of hospitalized patients who received any component of the composite outcome by the month of index hospitalization to examine seasonality, trends, and outlying values. The piecewise GLMM will be used to model the composite outcome. The primary explanatory variable will be a dichotomous variable for the time period (preintervention vs postintervention), and the covariates will include age, sex, race/ethnicity, and payor status. This model will take the form of:




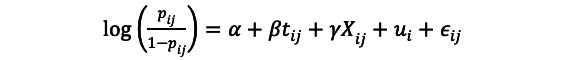




where *p_ij_* is the probability of the composite outcome for patient *i* at hospitalization *j, α* is the overall intercept, *β* is the coefficient for time period, *X_ij_* is the design matrix of covariates, *u_i_* is the patient-level random intercept, and *
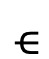
_ij_* is the random error term. Additional variables will be considered including season/month, primary diagnosis, and the unit, with fit statistics (Akaike information criterion [AIC] and Bayesian information criterion [BIC]) used to guide model selection. A similar GLMM will be specified to predict rehospitalization for the secondary end point.

For the exploratory end point of time to consult, among the subset receiving the composite outcome, a Poisson mixed-effects regression model will regress time to consult on time period (preintervention vs postintervention) and covariates. The rate of discharge AMA will be modelled similarly to the primary end point with mixed-effects logistic regression. Finally, the addition of interaction terms to the primary GLMM (eg, payor by intervention period) will be performed to test if some subgroups of patients may be more or less likely to experience the composite end point after the implementation of SMART-AI.

Our hospitalization-level analysis plan assumes the degree of seasonality and autocorrelation in this design will be minimal. If the assumption holds true, mixed-effects regression, which is flexible regarding outcome variable distribution and can account for both fixed effects (covariates) and random effects (nesting), is preferable to time-series approaches, which directly model the aggregated data. We believe this assumption is reasonable as we found a white noise model using 3 years of prior SUIT data (August 2018 to July 2020), which demonstrated negligible autocorrelation (AR_1,1_=0.04, SD 0.22; *P*=.86) and required no differencing to achieve stationarity (constant mean and constant variance over time). We will verify whether this assumption holds for the study period, and an interrupted time-series approach using autoregressive integrated moving average (ARIMA) models will be applied should autocorrelation be substantial [28]. In this case, a transformation will be applied to stabilize the variance over time if the data are heteroscedastic. Differencing will be applied to induce stationarity, such as a difference to account for linear trend (d=1, degree of nonseasonal differencing), and autocorrelation functions (ACFs) will be plotted to verify stationarity. After differencing, ACFs and partial ACFs will be used to determine the orders of autoregression or moving average that will correct the remaining autocorrelation. We will formally test if the intervention will promote a step change (binary indicator for a level shift when intervention begins) and a ramp effect (the variable takes the value of 0 before the intervention and increases by 1 for each month following the beginning of the intervention). Fit statistics (AIC and BIC) and residual analysis (Ljung-Box test for white noise) will be used to identify a parsimonious and appropriate model based on ARIMA orders, differencing assumptions, and parameters for intervention impact (step, pulse, or ramp).

### Cost-Benefit Analysis

In the cost-benefit analysis, both costs and consequences of alternatives are measured in monetary units [29]. We will conduct in-depth interviews with SUIT personnel and brief interviews with RUMC staff (nurses and social workers) to query about the fixed and variable costs associated with establishing and implementing substance misuse screening during the usual care with manual screening and AI-assisted screening periods. During both periods, we will ask about the time cost of staff receiving training and administering the universal screening (not applicable during the intelligence-assisted screening period), secondary screening, and brief intervention/motivational interviewing for unhealthy substance use, which is not a billable service within a hospitalization context. The time cost of staff and resources dedicated to building EHR infrastructure to support manual and AI-assisted screenings will also be calculated (Multimedia Appendix 1). Costs associated with the index hospitalization, including naloxone dispensing, MAT, other treatments, and same-hospital rehospitalizations, will be extracted from the EHR and administrative billing records. The cost-benefit analysis will be conducted using the health system perspective and within the hospitalization episode. We will use a mixed-effects generalized linear model with log link function and gamma distribution to calculate the adjusted cost or saving per index hospitalization during the preintervention and postintervention periods. Our analysis will adjust for patient-level sociodemographic and clinical characteristics and include random intercepts to account for multiple index hospitalizations per patient. We will repeat the analysis with and without the cost of same-hospital rehospitalizations. Results from the regression analysis will be combined with data collected from the interviews to calculate the incremental cost or saving per index hospitalization receiving substance misuse screening and the incremental cost or saving per index hospitalization receiving any component of the composite outcome during the manual screening period compared to the AI-screening period. We will also calculate the average cost of the SBIRT program per patient and the average cost of composite outcome per patient during each intervention period. The Hospital Care component of the Personal Health Care Price Index published by the Centers for Medicare and Medicaid Services will be used to adjust costs to analyze cost per year in US dollars [30].

### Sample Size Calculations

**Figure 1:**
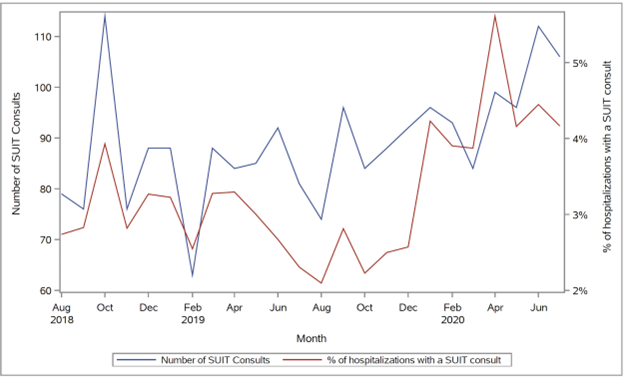
Monthly SUIT consults from 2018-2020. SUIT: Substance Use Intervention Team.

**Figure 2:**
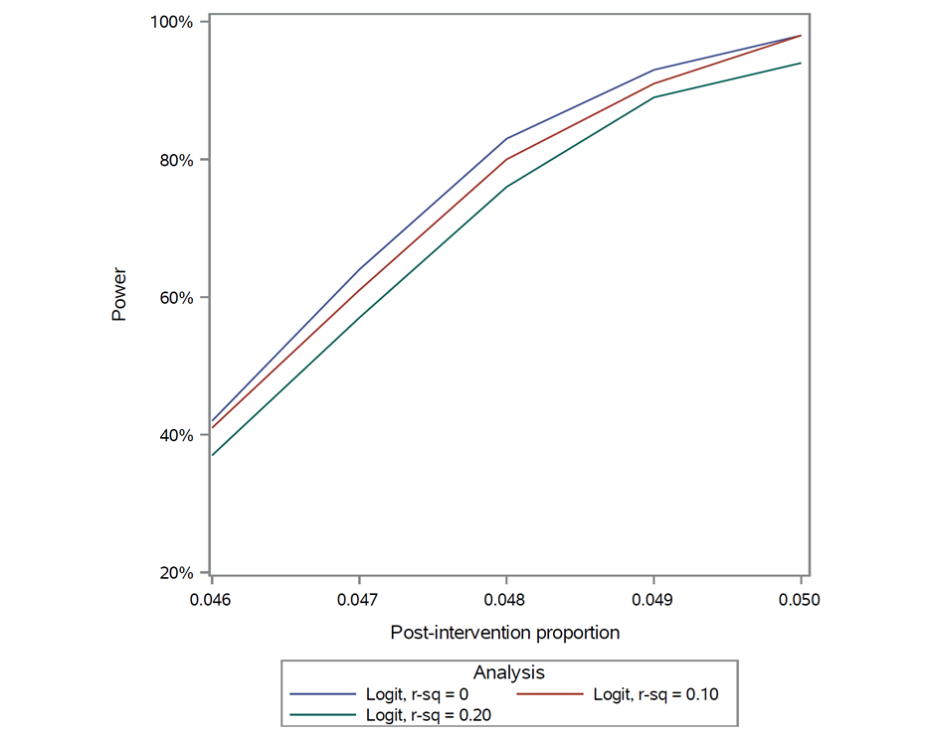
Power curve for covariate-adjusted logistic regression analysis. r-sq: R-squared.

In 2020, on average, approximately 2400 (SD 250) patients were hospitalized each month with 94 (SD 9) SUIT consults performed at RUMC. Overall, a median of 3.9% (IQR 3.5%-4.2%) of hospitalized patients received a SUIT consult during each month of 2020 (Figure 1). This period of time was chosen to inform power as it represents a “new normal” for SUIT practices in the era of COVID-19. We hypothesize that additional components of the composite outcome will lead to a new preintervention outcome rate of 4.8% of hospitalized patients. For the primary end point, the null hypothesis is that the difference in the proportion receiving the composite outcome *P*_2_ (postintervention) and *P*_1_ (preintervention) is less than or equal to a noninferiority difference *D*0 of 0.5% such that *H*_0_: *P*_2_ – *P*_1_ ≤ *D*0. This corresponds to the SMART-AI time period intervention rate of 4.3% or less under the null hypothesis of inferiority. A total sample size of 60,000, or 30,000 hospitalizations per time period, will have 82% power to detect a 0% difference using a 1-sided *Z* test and α=.025 (Table 2). Power is slightly attenuated in the setting for mixed-effects logistic regression with covariates compared to the simple *Z* test; however, we expect a minimal correlation between time period and covariates, or *R*-squared close to zero (Figure 2).

**Table 2:** Power estimates for a noninferiority hypothesis^a^.

Setting	Proportion with outcome		Noninferiority difference	Difference at which power is calculated	Power for 2 proportions
	Preintervention	Postintervention			
1	0.048	0.046	–0.005	–0.002	41%
2	0.048	0.047	–0.005	–0.001	63%
3	0.048	0.048	–0.005	0.000	82%
4	0.048	0.049	–0.005	0.001	93%
5	0.048	0.050	–0.005	0.002	98%

^a^Assumptions: α=.025, n=60,000 (50% per time period).

### Ethical Considerations

The project was considered secondary research, for which a waiver of consent was approved by the RUMC IRB on May 10, 2022.

## Results

**Figure 3:**
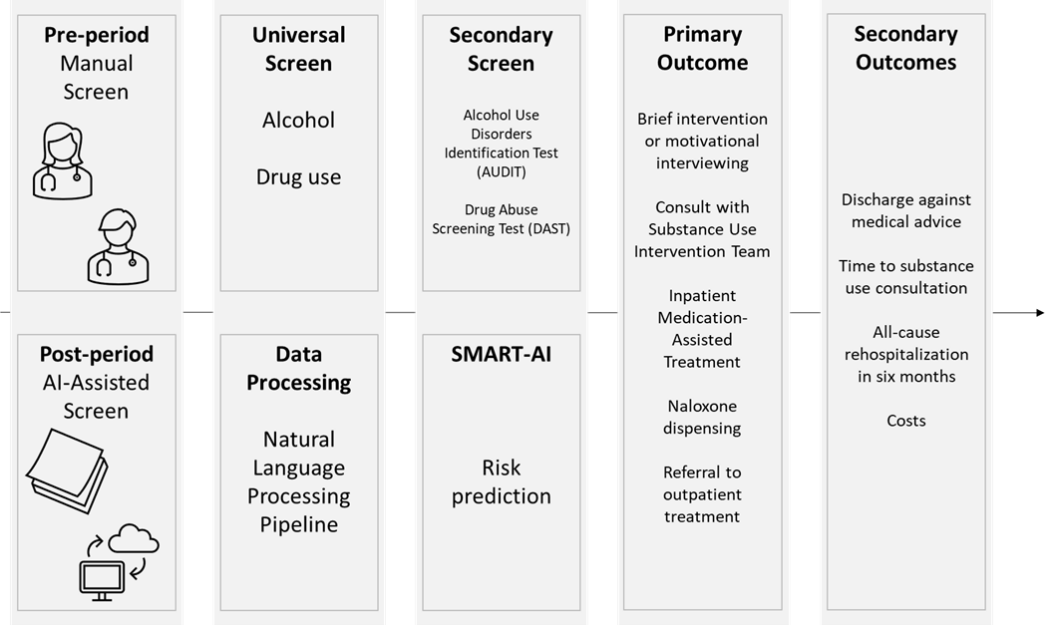
Study components and outcomes. AI: artificial intelligence. SMART-AI: Substance Misuse Algorithm for Referral to Treatment Using Artificial Intelligence.

The methods of screening and outcomes are summarized by time period in Figure 3. The preintervention study period will formally begin in September 2022 after a new round of education and in-servicing on screening to social workers and nurses for the manual screening phase. Monthly data monitoring and DSMB reporting are scheduled every 6 months throughout the study period. We anticipate reporting final results by June 2025.

## Discussion

There remains a paucity of protocols for evaluating AI systems in health care, because deploying such systems in large, complex health systems is relatively new [31]. Many AI models that are published and validated using retrospective data never reach the implementation phase for bedside evaluation [32]. Herein, we provide a protocol incorporating an automated substance use screening tool into an established screening program with the intent to improve throughput and efficiency over manual procedures. Our use case is an example of an AI system intended to improve efficiency and throughput within a reasonable time frame for hospital operations. In these cases, statistically superior performance on outcomes may not be expected or required for prospective implementation, and interventions may be desirable if they are both substantially equivalent (noninferior) on clinical outcomes and cost-effectiveness, given the high cost of building IT infrastructure and hiring vendors with high costs in licensing and software support. Our protocol provides one of the first use cases of NLP in CDS with AI-assisted screening for unhealthy substance use.

To our knowledge, no systems currently exist for AI-assisted screening of unhealthy substance use in health care settings. Screening rates for substance use disorders in health care systems remain low with many missed opportunities for care interventions, especially in emergency department settings where the prevalence of unhealthy substance use is high [33]. Further, comprehensive screening programs are needed to better understand the epidemiology and morbidity of substance use–related conditions. The total annual estimated attributable medical cost in patients with substance use–related admissions is US $13.2 billion, including US $7.6 billion from alcohol-related disorders alone [34]. The cost-effectiveness of treatment for hospitalized patients with substance use disorders has been described [21,35], but the role of an AI-assisted screening approach to further improve screening efforts and how it translates to health outcomes and cost remains unknown.

Past work on AI health systems has surfaced the following obstacles in going from research and development environments into clinical settings: (1) culture/personnel, (2) clinical utility of AI tools, (3) financing, (4) technology support, and (5) adequate data [36]. We provide an in-house solution by working with our data science team to develop a novel screening tool to address the priority of hospital-wide screening. The clinical utility of the tool and return on investment will be examined in our primary outcomes. Additional challenges remain in information systems discovery and program management with clinical champions and executive sponsorship to help align with institutional business needs. Using open-source software for NLP processing and following best practices in the model evaluation should help keep costs low. However, costs in the cloud computing platform with Health Level Seven (HL7) standards and EHR vendor integration have limited our implementation to a fully integrated CDS that is embedded directly into the EHR. Less costly steps to leverage existing data warehouse capabilities are currently planned as an alternative to the integrated EHR workflow to provide daily screening reports to the care team. Ultimately, this may affect the process measures and, in turn, proposed outcomes.

More protocols are needed describing AI-assisted CDS tools for hospital implementation with an evaluation framework that is conducive to system-wide implementation. Although conventional parallel-group randomized controlled trials may be considered the gold standard for evaluation, they are costly and require substantial external resources to be implemented. Alternatives such as the stepped wedge cluster randomized trial offer operational efficiency and some cost reductions [37] but can introduce new biases and require larger sample sizes to achieve similar power. When randomized trials are not feasible due to available resources, carefully selected quasi-experimental designs provide good alternatives for evaluation. Without randomization, these designs have limitations including the potential for bias due to secular trends and confounders, which may only partially be controlled for analytically. Additionally, with widespread implementation across a large hospital, any single condition or disease contributes to a low prevalence of cases monthly and may prove difficult to evaluate effectively. A low case rate may limit statistical power in analytic approaches such as the interrupted time-series design [38].

This protocol follows best practices in reporting our AI system and implementation approach, with an evaluation framework on large-scale effectiveness [39]. In addition, we have an established DSMB to also provide oversight into safety and ethics. We are meeting some of the core components of the Quadruple Aim to enhance health care efficiency [40]. Reducing costs and improving population health are the components we address, but our protocol is limited in examining other aims such as patient experience and provider well-being. Future work should include protocols incorporating the other components of the Quadruple Aims for optimizing health care delivery.

We attempt to minimize limitations in the pre-post design by using a well-powered but short time frame to minimize secular trends and by collecting extensive patient characteristics to control for potential changes to the demographics of our target population over time. Nevertheless, limitations of our pragmatic study include disruptions in hospital staffing to perform the consultations recommended by the AI system and that threaten the fidelity of the automated screening. In addition, unpredicted secular trends may occur and introduce additional confounding into the study or disruptions in health IT services to maintaining and updating the software dependencies for the AI infrastructure. Alternative strategies include incorporating implementation frameworks into the study protocol that are capable of achieving a rapid plan-do-study-act cycle to meet the operational needs of the health system and minimize disruptions, so that appropriate evaluation of the AI system’s effectiveness may be achieved.

The successful implementation of the SMART-AI screening tool in hospitalized patients is a step toward an automated and comprehensive universal screening system for unhealthy substance use. We expect our results to demonstrate that the automated screener will increase the proportion of hospitalized patients with unhealthy substance use who screen positive and receive a brief intervention or referral to treatment. The dissemination of the expected results from this research would allow standardized and scalable “NLP-capable” measures for health care systems to identify patients with unhealthy substance use.

## Appendix

Multimedia Appendix 1 Overview of data elements for the cost-benefit analysis.

## Abbreviations

ACF: autocorrelation function
AI: artificial intelligence
AIC: Akaike information criterion
AMA: against medical advice
ARIMA: autoregressive integrated moving average
AUDIT: Alcohol Use Disorders Identification Test
BIC: Bayesian information criterion
CDS: Clinical decision support
CNN: convolutional neural network
cTAKES: Clinical Text and Knowledge Extraction System
CUI: Concept Unique Identifier
DAST: Drug Abuse Screening Tool
DSMB: Data Safety Monitoring Board
EHR: electronic health record
GLMM: generalized linear mixed effects model
HL7: Health Level Seven
MAT: medication-assisted treatment
NLP: natural language processing
NNE: number needed to evaluate
RUMC: Rush University Medical Center
SBIRT: Screening, Brief Intervention, and Referral to Treatment
SMART-AI: Substance Misuse Algorithm for Referral to Treatment using Artificial Intelligence
SUIT: Substance Use Intervention Team
UMLS: Universal Medical Language System

## Data Availability

Data sharing is not applicable to this publication as this is a protocol paper and data collection has not begun.

## References

[1] Brown KG, Chen CY, Dong D, Lake KJ, Butelman ER (2022). Has the United States reached a plateau in overdoses caused by synthetic opioids after the onset of the COVID-19 pandemic? examination of Centers for Disease Control and Prevention data to November 2021. Front Psychiatry.

[2] Owens PL, Fingar KR, McDermott KW, Muhuri PK, Heslin KC (2019). Inpatient stays involving mental and substance use disorders, 2016. Healthcare Cost and Utilization Project (HCUP) Statistical Briefs.

[3] Krist Alex H, Davidson Karina W, Mangione Carol M, Barry Michael J, Cabana Michael, Caughey Aaron B, Curry Susan J, Donahue Katrina, Doubeni Chyke A, Epling John W, Kubik Martha, Ogedegbe Gbenga, Pbert Lori, Silverstein Michael, Simon Melissa A, Tseng Chien-Wen, Wong John B, US Preventive Services Task Force (2020). Screening for unhealthy drug use: US Preventive Services Task Force Recommendation Statement. JAMA.

[4] Serowik KL, Yonkers KA, Gilstad-Hayden K, Forray A, Zimbrean P, Martino S (2021). Substance use disorder detection rates among providers of general medical inpatients. J Gen Intern Med.

[5] White AM, Slater ME, Ng G, Hingson R, Breslow R (2018). Trends in alcohol-related emergency department visits in the United States: results from the Nationwide Emergency Department Sample, 2006 to 2014. Alcohol Clin Exp Res.

[6] Schulte MT, Hser Y (2013). Substance use and associated health conditions throughout the lifespan. Public Health Rev.

[7] Park-Lee E, Lipari RN, Hedden SL, Kroutil LA, Porter JD (2017). Receipt of services for substance use and mental health issues among adults: results from the 2016 National Survey on Drug Use and Health. CBHSQ Data Review.

[8] Afshar M, Sharma B, Dligach D, Oguss M, Brown R, Chhabra N, Thompson HM, Markossian T, Joyce C, Churpek MM, Karnik NS (2022). Development and multimodal validation of a substance misuse algorithm for referral to treatment using artificial intelligence (SMART-AI): a retrospective deep learning study. Lancet Digit Health.

[9] Fingar KR, Barrett ML, Jiang HJ (2017). A comparison of all-cause 7-day and 30-day readmissions, 2014. Healthcare Cost and Utilization Project (HCUP) Statistical Briefs.

[10] Adler-Milstein J, Holmgren A, Kralovec P, Worzala C, Searcy T, Patel V (2017). Electronic health record adoption in US hospitals: the emergence of a digital "advanced use" divide. J Am Med Inform Assoc.

[11] Lite S, Gordon WJ, Stern AD (2020). Association of the meaningful use electronic health record incentive program with health information technology venture capital funding. JAMA Netw Open.

[12] Maletzky A, Böck Carl, Tschoellitsch T, Roland T, Ludwig H, Thumfart S, Giretzlehner M, Hochreiter S, Meier J (2022). Lifting hospital electronic health record data treasures: challenges and opportunities. JMIR Med Inform.

[13] Liu N, Xie F, Siddiqui FJ, Ho AFW, Chakraborty B, Nadarajan GD, Tan KBK, Ong MEH (2022). Leveraging large-scale electronic health records and interpretable machine learning for clinical decision making at the emergency department: protocol for system development and validation. JMIR Res Protoc.

[14] Hsu M, Ahern DK, Suzuki J (2020). Digital phenotyping to enhance substance use treatment during the COVID-19 pandemic. JMIR Ment Health.

[15] Palumbo SA, Adamson KM, Krishnamurthy S, Manoharan S, Beiler D, Seiwell A, Young C, Metpally R, Crist RC, Doyle GA, Ferraro TN, Li M, Berrettini WH, Robishaw JD, Troiani V (2020). Assessment of probable opioid use disorder using electronic health record documentation. JAMA Netw Open.

[16] Chartash D, Paek H, Dziura JD, Ross BK, Nogee DP, Boccio E, Hines C, Schott AM, Jeffery MM, Patel MD, Platts-Mills TF, Ahmed O, Brandt C, Couturier K, Melnick E (2019). Identifying opioid use disorder in the emergency department: multi-system electronic health record-based computable phenotype derivation and validation study. JMIR Med Inform.

[17] Barenholtz E, Fitzgerald ND, Hahn WE (2020). Machine-learning approaches to substance-abuse research: emerging trends and their implications. Curr Opin Psychiatry.

[18] Smothers BA, Yahr HT (2005). Alcohol use disorder and illicit drug use in admissions to general hospitals in the United States. Am J Addict.

[19] Sheikhalishahi S, Miotto R, Dudley JT, Lavelli A, Rinaldi F, Osmani V (2019). Natural language processing of clinical notes on chronic diseases: systematic review. JMIR Med Inform.

[20] Lederman A, Lederman R, Verspoor K (2022). Tasks as needs: reframing the paradigm of clinical natural language processing research for real-world decision support. J Am Med Inform Assoc.

[21] Thompson HM, Faig W, VanKim NA, Sharma B, Afshar M, Karnik NS (2020). Differences in length of stay and discharge destination among patients with substance use disorders: the effect of Substance Use Intervention Team (SUIT) consultation service. PLoS One.

[22] Reinert DF, Allen JP (2002). The Alcohol Use Disorders Identification Test (AUDIT): a review of recent research. Alcoholism Clin Exp Res.

[23] Yudko E, Lozhkina O, Fouts A (2007). A comprehensive review of the psychometric properties of the Drug Abuse Screening Test. J Subst Abuse Treat.

[24] Apache cTAKES.

[25] (2022). SMART-AI.

[26] Tran TH, Swoboda H, Perticone K, Ramsey E, Thompson H, Hill K, Karnik Niranjan S (2021). The substance use intervention team: a hospital-based intervention and outpatient clinic to improve care for patients with substance use disorders. Am J Health Syst Pharm.

[27] Thompson H, Hill K, Jadhav R, Webb T, Pollack M, Karnik N (2019). The Substance Use Intervention Team: a preliminary analysis of a population-level strategy to address the opioid crisis at an academic health center. J Addict Med.

[28] Hyndman RJ, Athanasopoulos G (2018). Forecasting: principles and practice. 2nd ed.

[29] Drummond M, Sculpher MJ, Claxton K, Stoddart GL, Torrance GW (2015). Methods for the Economic Evaluation of Health Care Programmes.

[30] Dunn A, Grosse SD, Zuvekas SH (2018). Adjusting health expenditures for inflation: a review of measures for health services research in the United States. Health Serv Res.

[31] Cresswell K, Sheikh A (2013). Organizational issues in the implementation and adoption of health information technology innovations: an interpretative review. Int J Med Inform.

[32] Zhou Q, Chen Z, Cao Y, Peng S (2021). Clinical impact and quality of randomized controlled trials involving interventions evaluating artificial intelligence prediction tools: a systematic review. NPJ Digit Med.

[33] Smith JR, Hazen EP, Kaminski TA, Wilens TE (2020). Literature review: substance use screening and co-morbidity in medically hospitalized youth. Gen Hosp Psychiatry.

[34] Peterson C, Li M, Xu L, Mikosz CA, Luo F (2021). Assessment of annual cost of substance use disorder in US hospitals. JAMA Netw Open.

[35] Barocas JA, Savinkina A, Adams J, Jawa R, Weinstein ZM, Samet JH, Linas BP (2022). Clinical impact, costs, and cost-effectiveness of hospital-based strategies for addressing the US opioid epidemic: a modelling study. Lancet Public Health.

[36] Marwaha JS, Landman AB, Brat GA, Dunn T, Gordon WJ (2022). Deploying digital health tools within large, complex health systems: key considerations for adoption and implementation. NPJ Digit Med.

[37] Hemming K, Taljaard M (2020). Reflection on modern methods: when is a stepped-wedge cluster randomized trial a good study design choice?. Int J Epidemiol.

[38] Zhang F, Wagner AK, Ross-Degnan D (2011). Simulation-based power calculation for designing interrupted time series analyses of health policy interventions. J Clin Epidemiol.

[39] Vasey B, Nagendran M, Campbell B, Clifton DA, Collins GS, Denaxas S, Denniston AK, Faes L, Geerts B, Ibrahim M, Liu X, Mateen BA, Mathur P, McCradden MD, Morgan L, Ordish J, Rogers C, Saria S, Ting DSW, Watkinson P, Weber W, Wheatstone P, McCulloch P, DECIDE-AI expert group (2022). Reporting guideline for the early stage clinical evaluation of decision support systems driven by artificial intelligence: DECIDE-AI. BMJ.

[40] Arnetz BB, Goetz CM, Arnetz JE, Sudan S, vanSchagen J, Piersma K, Reyelts F (2020). Enhancing healthcare efficiency to achieve the Quadruple Aim: an exploratory study. BMC Res Notes.

