# Supplementary material for: The Evaluation of a Clinical Decision Support Tool Using Natural Language Processing to Screen Hospitalized Adults for Unhealthy Substance Use: Protocol for a Quasi-Experimental Design
Source: JMIR Res Protoc. 2022 Dec 19;11(12):e42971. doi: 10.2196/42971 (PMC9808720; doi:10.2196/42971)
Supplement: Multimedia Appendix 1 [file resprot_v11i12e42971_app1.docx]

| Appendix 1. Overview of data elements for the Cost-Benefit Analysis | | |
| --- | --- | --- |
| Activities | **Usual care with manual screening** | **Artificial intelligence (AI)-assisted screening** |
| **Fixed costs: Intervention establishment phase** | | |
| Building and administering intervention associated trainings (as needed) | Time cost^a^ of trainers (program personnel) and trainees (social workers, nurses, and clinicians) | Time cost^a^ of trainers (program personnel) and trainees (social workers, nurses, and clinicians) |
| Building Electronic Health Record (EHR) infrastructure to support screening | Time cost^a^ of program personnel and EHR technicians | Time cost^a^ of program personnel and EHR technicians |
| Other intervention related support activities | Time cost^a^ of program personnel | Time cost^a^ of program personnel |
| **Variable costs: Program implementation phase**  **[Costs are multiplied by number of patients receiving the service]** | | |
| Universal screen | Time cost^a^ of administering universal screening by type of staff (social work or nurse) | N/A |
| Full screen by AUDIT and DAST | Time cost^a^ of administering full screening by type of staff (social work or nurse) | Time cost^a^ of administering full screening by type of staff  (social work or nurse) |
| Brief Intervention (BI)/motivational interviewing (MI) | Time cost^a^ of administering BI/MI by type of staff (social work or nurse) | Time cost^a^ of administering BI/MI by type of staff (social work or nurse) |
| Index hospitalization | Cost of service^b^ | Cost of service^b^ |
| Naloxone dispensing | Cost of service^b^ | Cost of service^b^ |
| Medication assisted treatment and other addiction associated treatments | Cost of service^b^ | Cost of service^b^ |
| Addiction related laboratory testing | Cost of service^b^ | Cost of service^b^ |
| Same-hospital re-hospitalization | Cost of service^b^ | Cost of service^b^ |

^a^Data collected from program personnel and staff (social work and nurse) interviews and/or direct observation.

^b^Data extracted from Electronic Health Record and administrative billing records.

SUIT, Screening, Brief Intervention, and Referral to Treatment; AUDIT, Alcohol Use Disorders Identification Test; DAST, Drug Abuse Screening Tool.
